# Protective Effects of Peroxisome Proliferator-Activated Receptor-**α** Agonist, Wy14643, on Hypoxia/Reoxygenation Injury in Primary Rat Hepatocytes

**DOI:** 10.1155/2012/547980

**Published:** 2011-10-09

**Authors:** Ke Chen, Yuan-Hai Li, Si-Qi Xu, Sheng-Hong Hu, Lei Zhang

**Affiliations:** ^1^Department of Anesthesiology, First Affliated Hospital of Anhui Medical University, Hefei 230022, China; ^2^Department of Pharmacology, Anhui Medical University, Hefei 230022, China

## Abstract

This study investigates the effects and possible mechanism of an agonist of PPAR**α**, Wy14643, on primary hepatocytes subjected to H/R injury in rats. H/R induced a significant increase ALT, AST, MDA in the culture medium and ROS in the hepatocytes. These effects were reversed by pretreatment with Wy14643 in the dose-dependent manner. The activity of SOD and the level of GSH in the hepatocytes were decreased after H/R, which were increased by Wy14643 pretreatment. Moreover, the mRNA expressions of PPAR**α** significantly increased in H/R+Wy14643 groups when compared with that in H/R group. A PPAR**α** agonist, Wy14643, exerts significant protective effect against H/R injury in primary hepatocytes via PPAR**α** activation and attenuating oxidative stress.

## 1. Introduction

Ischemia/reperfusion (I/R) injury is a serious complication precipitated during short-term expansion of the invading parasitic pathogens, such as by* Entamoeba histolytica *as the infections lead to local tissue damage and hypoxia [1]. The condition has also been reported to occur in certain intracellular bacterial infections, for example, *Chlamydia *species. Mechanisms of I/R injury involve complex and multiple pathways, including the direct ischemic cellular damage as well as the cell injury due to the activation of inflammatory response after reperfusion [2]. A hepatic, in vitro hypoxia/reoxygenation (H/R) model can be use to study the pathophysiology of this injury [3]. Previous study showed that the generation of reactive oxygen species (ROS) is likely to be an important factor in H/R-induced cell damage. Thus, ROS are generated immediately after H/R and activate proapoptotic/inflammatory signalling in the cell or directly damage cell organelles. These include direct oxidation of cellular components and lipids (lipid peroxidation), activation of inflammatory gene transcription, and possible activation of the innate immune response [2, 3]. Administration of antioxidants such as glutathione could afford protection against I/R injury [4, 5]. Oxygen deprivation (hypoxia) during ischemia and subsequent reoxygenation upon reperfusion are thought to be the major factors contributing to ROS production and the subsequent cellular damage [6]. Hypoxia increases mitochondrial reactive oxygen species (ROS) generation at Complex III, and the predominant source of ROS by oxygen limitation originates from mitochondria [7]. Peroxisome proliferator-activated receptors (PPARs) are members of the nuclear receptor related to retinoid, steroid, and thyroid hormone receptors. Peroxisome proliferator-activated receptor-*α* (PPAR*α*) is one of the three subtypes of the nuclear receptor PPAR family [8]. The structure of PPAR*α* consists of an aminoterminal region that allows for ligand-independent activation and constitutive activity on the receptor and is negatively regulated by phosphorylation and the carboxyl-terminal ligand binding domain. PPAR*α* has a wide range of effects on metabolism, cellular proliferation and the immune response [9]. Besides its metabolic regulating effects, PPAR*α* also exerts anti-inflammatory and antioxidant effects in different organs. As is well known that PPAR*α* have anti-inflammatory properties [10], we proposed that it may have a similar effects on hepatic I/R injury. Previous studies suggest that PPAR*α* agonists protect many organs against I/R injury such as heart [11], kidney [12], and brain [13]. PPAR*α* agonists can inhibit the expression of oxidative stress genes via a mechanism termed ligand-dependent repression by PPAR*α*. It has been demonstrated that the anti-inflammatory effect of PPAR*α* ligands is also dependent on the inhibition of functional NF-*κ*B activation and AP-1 activation [14]. Furthermore, PPAR*α* stimulation by Wy14643 induces expression and activation of antioxidant enzymes such as superoxide dismutase (SOD), catalase, and glutathione (GSH), which protects hepatocytes against hepatic I/R injury mice model in vivo [15]. These beneficial effects of Wy14643 are possibly associated with enhancement of anti-oxidant and inhibition of inflammation response. Razeghi et al. have found a downregulation in expression of PPAR*α* in a rat model of hypoxia [16]. However, there is no report about the effect of H/R on expression of hepatocytes PPAR*α*-mRNA in vitro. In the present study, we determined whether PPAR*α* activation by the selective agonist Wy14643 had a protective role in H/R injury of hepatocytes in vitro.

## 2. Materials and Methods

### 2.1. Animals

Male Sprague-Dawley rats (weighing 220–280 g) were used in these experiments. Temperature and relative humidity were kept at (22 ± 2)°C and (50 ± 5)%, respectively. All rats were obtained from the Center of Experimental Animals in Anhui Medical University. This project was approved by the Committee for Research and Animal Ethics of the Anhui Medical University. They were allowed free access to a commercial standard chow and water ad libitum before the experimental procedure began. All rats were acclimatized to our animal facility for at least 1 week before experiment to avoid stressful stimuli.

### 2.2. Hepatocyte Isolation and Culture

Rat hepatocytes were isolated and cultured as previously described [17]. Male Sprague-Dawley rat livers were minced after perfusion with 0.5 g/L collagenase IV (Sigma, USA) through the portal vein. Hepatocytes were separated from nonparenchymal cells by centrifugation at 50 g for 4 min at 4°C. The viability of the collected hepatocytes exceeded 90%, as determined by the trypan blue exclusion test. Hepatocytes were resuspended in William's E medium (Gibco, USA) containing 100 mL/L fetal calf serum, 27 × 10^−6^ mol/L NaHCO_3_, 100 × 10^−9^ mol/L insulin, and 10 × 10^−9^ mol/L dexamethasone at pH 7.4. Hepatocytes were then plated on type I collagen—coated 24—multiwell plates and incubated overnight in an atmosphere of 95% air and 5% CO_2_ at 37°C. Cells were studied according to the experimental protocols.

### 2.3. Hypoxia/Reoxygenation Treatments and Groups

H/R injury in vitro was performed as previously described [18]. Hepatocytes were isolated and maintained overnight at 37°C in a humidified incubator containing 95% air and 5% CO_2_ (referred to as normoxic conditions). The next day, hypoxic conditions were attained by exposure to 95% N_2_ and 5% CO_2_ gas mixture in a humidified incubator for 4 h. Hypoxic exposure was confirmed in each experiment by measuring the ambient PO_2_ of the gas above the monolayers. Reoxygenation of hypoxic cultures was achieved with normoxia conditions for another 10 h, whose ambient values should return to pre-hypoxic levels within 5 min. Six groups of culture hepatocytes (6 wells each) were separated as follows. (1) The control group was exposed to normoxic medium for 14 h. (2) The H/R injury group was exposed to hypoxic (4 h) and then reoxygenation (10 h) conditions as described above. (3) Model H/R hepatocytes treated with different doses of Wy14643 (10 × 10^−6^, 30 × 10^−6^, and 100 × 10^−6^ mol/L, resp.) (Pirinixic acid, CAS 50892-23-4, Cayman Chemical, USA). Different concentrations of Wy14643 were added to the culture medium 60 min before H/R course [15]. Wy14643 were prepared in 10% (v/v) DMSO (dimethyl sulfoxide) and 90% (v/v) William's E medium. The final DMSO concentration in cell cultures was 0.1% (this concentration affected neither cell viability nor hepatocytes damage). (4) DMSO group hepatocytes were pretreated for 60 min with 0.1% DMSO before H/R.

### 2.4. Mitochondria Isolation

The cell medium was collected, centrifuged at 450 g to remove cell debris. The hepatocytes mitochondrial fraction was prepared according to the method reported by Johnson and Lardy. The homogenate was centrifuged at 600 g for 10 min, and the supernatant was centrifuged for 5 min at 15,000 g to obtain the mitochondrial pellet which was washed with a medium and centrifuged for 5 min at 15,000 g.

### 2.5. Measurement of Intracellular ROS Generation

2,7-Dichlorofluorescein diacetate (DCFH-DA) was used as indicatior of intracellular formation of ROS as described previously [19]. DCFH-DA is cell-permeant probe that enters the cell followed by cleavage of the diacetate molecules by cellular esterases. The probe becomes fluorescent when it is oxidized in cells by ROS. In brief, hepatocytes were plated onto 24-well plates at a density of 60,000 cells/well. 24 h after plating, cells were washed twice with PBS and subsequently incubated in PBS+ containing 10 *μ*M DCHFH-DA (Invitrogen, Calif, USA) for 45 min at dark. Subsequently, cells were rinsed with PBS, and 500 *μ*L of fresh Earle's salt solution was added to each well. Fluorescence was measured using prewarmed SpectraMax (Molecular Devices, Calif, USA) spectrofluorometer, with excitation wavelengths of 485/535 nm for 20 min. The slope of the linear part of the graph was used to calculate the rate of increases in fluorescence. As a positive control, H_2_O_2_ was added just before placing the plate into the plate reader. The increase in fluorescence by ROS production was expressed as % of control.

### 2.6. Detection of Aminotransferase (ALT), Aspartate Aminotransferase (AST), and Malondialdehyde (MDA) Level in Culture Medium

The Lipid peroxidation in cultured hepatocyte was determined by detecting the level of MDA, which is the end product of lipid peroxidation; in the liver mitochondria was determined by measuring the level of the thiobarbituric acid-reactive substances spectrophotometrically at 532 nm according to the method reported by Buege and Aust. ALT and AST, markers of hepatocellular injury, were measured using commercial available kit. These assay kits were purchased from the Jiancheng Bioengineering Institute (Jiancheng, China) [20].

### 2.7. Measurement of Glutathione (GSH) Levels and Activity of Supeoxide Dismutase (SOD) in the Hepatocytes

GSH content was measured by the Owens and Belcher method, which was determined using the following procedure. The isolated mitochondria were suspended in a buffer (pH 7.5) containing 0.1 mol/L sodium phosphate and 5 mmol/L EDTA. After precipitating with 15% (w/v) sulfosalicylic acid containing 5 mmol/L EDTA, the total glutathione level was measured spectrophotometrically at a wavelength of 412 nm using yeast glutathione reductase and 5,50-dithio-bis(2-nitrobenzoic acid), as described by Tietze. SOD activity was measured through the inhibition of nitroblue tetrazolium (NBT) reduction by O_2_ generated by the xanthine oxidase system. The content of GSH and activity of SOD were measured using commercial kits (Jiancheng, China) [21].

### 2.8. Ultrastructure Assessment of Rat Hepatocytes

Hepatocytes fixed in 10 g/L Glutaral, dehydrated, dried and surface gilded according to standard procedures. Electron microscope was used to assess the degree of hepatic damage (JEM-1010, JEOL, Japan).

### 2.9. Measurement of PPAR*α* mRNA Levels by Real-Time Polymerase Chain Reaction (Real Time PCR)

Total RNA was extracted from cultured hepatocytes as recently described. Total RNA was extracted using the Trizol reagent (Invitrogen, USA), the value A at 260/280 nm was detected, and the concentration of RNA was calculated. cDNA was synthesized according to the manufacturer's instruction for the reverse transcription kit (Promega, USA), then semiquantitative real-time polymerase chain reaction analysis using SYBR Green PCR Master Mix (Invitrogen, USA). The cDNAs were quantified with an ABI StepOne sequence detection system (Applied Biosystems, Calif, USA). Primers were synthesized by Sangon (China). The rat, specific primer (sense and antisense primers) for PPAR*α* was sense: 3′-GTGGCTGCTATAATTTGCTGTG-5′, antisense: 5′-GAAGGTGTCATCTGGATGGGT-3′. The primer *β*-action was sense: 5′-TGGAATCCTGTGGCATTCCATCCATGAAAC-3′, antisense: 5′-AGGCTATCCCAGGCTTTGC-3′. Real-time PCR was performed in a 25 *μ*L of reaction containing 12.5 *μ*L of 2X SYBR Green Supermix, 200 nM primers and cDNA. The cycles for PCR were as follows: 95°C for 20 s, 54°C for 7 min, 40 cycles of 95°C for 20 s, 54°C for 30 s, and 72°C for 30 s. The fold changes in gene expression of PPAR*α* was calculated using the comparative *C*_*t*_ (cross-threshold) method. Briefly the *C*_*t*_ of the housekeeping gene *β*-action was subtracted from the *C*_*t*_ of PPAR*α* to get Δ*C*_*t*_. The Δ*C*_*t*_ value of control sample was the subtracted Δ*C*_*t*_ of the rest of the treatments to get the ΔΔ*C*_*t*_ value. Fold differences compared to control sample are obtained by calculating 2^−ΔΔ*C*_*t*_^ for each treatment group.

### 2.10. Statistical Analyses

All data were expressed as mean ± SD. The statistical significance of differences between groups was analyzed using the one-way analysis of variance (ANOVA) and methods of LSD with the SPSS11.5 for windows XP statistical software package. The *P* values less than 0.05 was considered statistically significant.

## 3. Results

### 3.1. Pretreatment with the PPAR*α* Agonist Wy14643 Decreases Hepatocytes Damage Induced by H/R Injury

**Figure 1:**
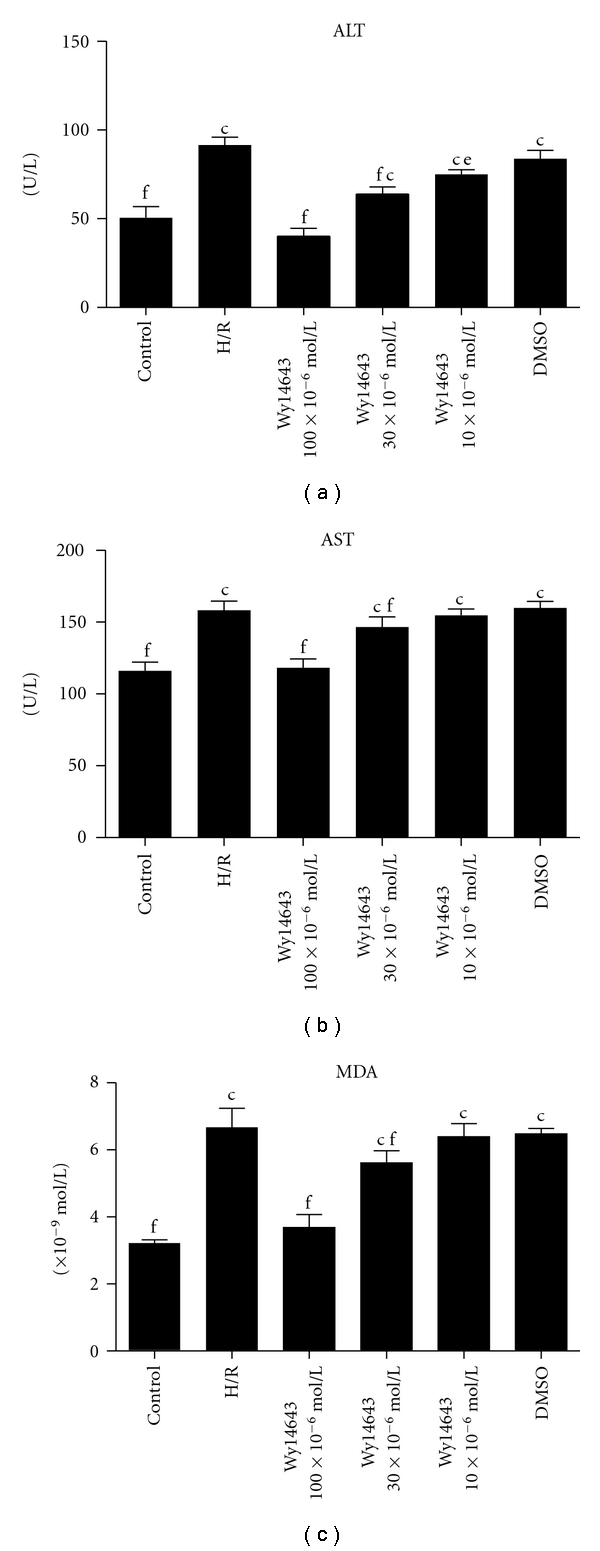
(a) ALT in different groups (Mean ± SD, *n* = 6). After 4 h of hepatocellular hypoxia and 10 h of reoxygenation. The levels of ALT in hepatocytes medium were determined. ^c^ *P* < 0.01 versus control group; ^e^ *P* < 0.05, ^f^ *P* < 0.01 versus H/R group. (b) AST in different groups (Mean ± SD, *n* = 6). After 4 h of hepatocellular hypoxia and 10 h of reoxygenation. The levels of AST in hepatocytes medium were determined. ^c^ *P* < 0.01 versus control group; ^f^ *P* < 0.01 versus H/R group. (c) MDA in different groups (Mean ± SD, *n* = 6). After 4 h of hepatocellular hypoxia and 10 h of reoxygenation. The levels of MDA in hepatocytes determined.^c^ *P* < 0.01 versus control group; ^f^ *P* < 0.01 versus H/R group.

ALT and AST levels in the medium of hepatocytes cultured under normal condition and H/R stress were shown in Figures 1(a) and 1(b), respectively. ALT and AST level increased after H/R (ALT, *P* = 0.006; AST, *P* = 0.0032). The increase in the medium ALT as well as AST activity induced by hepatic H/R was significantly attenuated by pretreatment Wy14643 (100 × 10^−6^, 30 × 10^−6^, 10 × 10^−6^ mol/L) in a dose-dependent manner. (ALT, *P* = 0.004; *P* = 0.095; *P* = 0.041. AST, *P* = 0.001; *P* = 0.0062; *P* = 0.0071.) DMSO group was not considered statistically when compared with H/R group (*P* = 0.08) the results demonstrated Wy14643 has the dose-dependent protective effects on hepatic injury.

### 3.2. Pretreatment with the PPAR*α* Agonist Wy14643 Increases Antioxidant Enzymes Induced by H/R Injury

**Figure 2:**
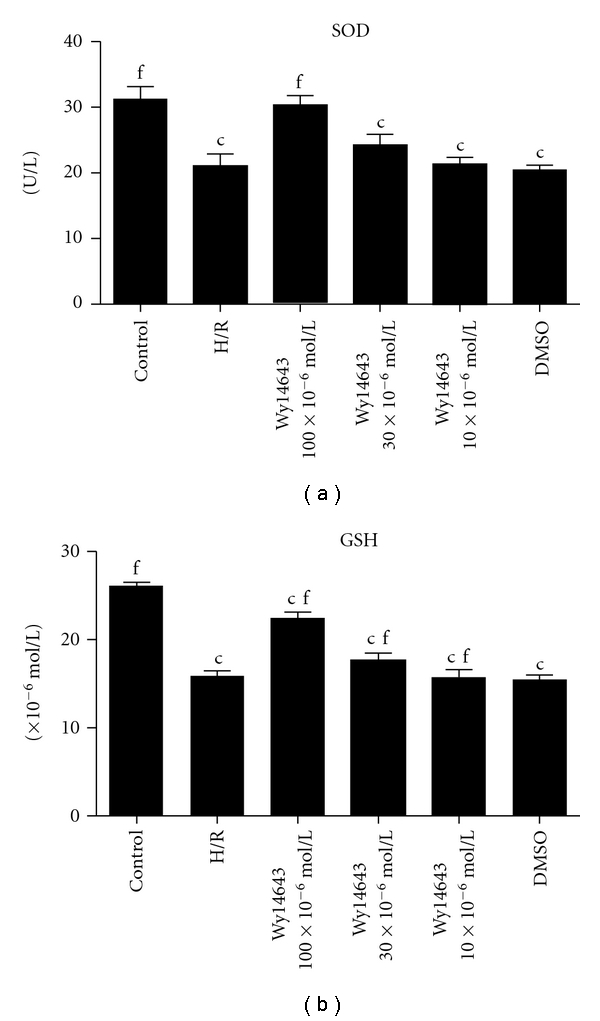
(a) SOD in different groups (Mean ± SD, *n* = 6). After 4 h of hepatocellular hypoxia and 10 h of reoxygenation. The levels of SOD in hepatocytes determined. ^c^ *P* < 0.01 versus control group; ^f^ *P* < 0.01 versus H/R group. (b) GSH in different groups (Mean ± SD, *n* = 6). After 4 h of hepatocellular hypoxia and 10 h of reoxygenation. The content of GSH in hepatocytes mitochondria fractions was determined. ^c^ *P* < 0.01 versus control group; ^f^ *P* < 0.01 versus H/R group.

The MDA level increased after H/R and maintained at a considerably high concentration during the period of cultivation (*P* = 0.008). Wy14643-pretreated group (100 × 10^−6^, 30 × 10^−6^, 10 × 10^−6^ mol/L) exhibited a decrease in the content of MDA compared with H/R group (*P* = 0.0025; *P* = 0.0094; *P* = 0.056) (Figure 1(c)). SOD and GSH were presented in Figure 2(a) and Figure 2(b). In H/R group, both these enzymes activities were significantly lower when compared with control group (SOD, *P* = 0.0003; GSH, *P* = 0.0005). But in the Wy14643-pretreated group (100 × 10^−6^, 30 × 10^−6^, 10 × 10^−6^ mol/L), the antioxidant activities were markedly higher when compared with the H/R group (SOD, *P* = 0.002; *P* = 0.059; *P* = 0.076. GSH, *P* = 0.0017; *P* = 0.0075; *P* = 0.0098). DMSO group was not considered statistically when compared with H/R group (*P* = 0.085).

### 3.3. Pretreatment with the PPAR*α* Agonist Wy14643 Decreases ROS Induced by H/R Injury

**Figure 3:**
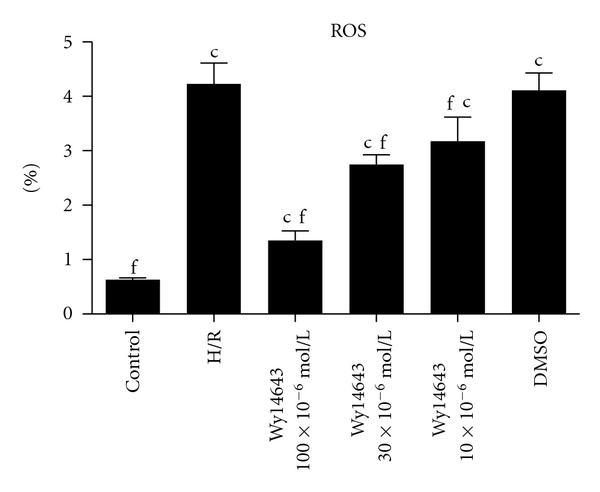
The content of ROS in different groups was determined by DCFH-DA (Mean ± SD, *n* = 6). After 4 h of hepatocellular hypoxia and 10 h of reoxygenation. ^c^ *P* < 0.01 versus control group; ^f^ *P* < 0.01 versus H/R group.

*ROS *levels in the hepatocytes cultured under normal condition and H/R stress were shown Figure 3. ROS level increased after H/R (ROS, *P* = 0.0032), The increase in the hepatocytes induced by hepatic H/R was significantly attenuated by pretreatment of Wy14643 (100 × 10^−6^, 30 × 10^−6^, 10 × 10^−6^ mol/L) in a dose dependent manner. (*P* = 0.0025; *P* = 0.0070; *P* = 0.0090). DMSO group was not considered statistically when compared with H/R group (*P* = 0.08); the results demonstrated Wy14643 has the dose-dependent decrease ROS on hepatic injury.

### 3.4. PPAR*α* Agonist Wy14643 Upregulates Hypoxia-/Reoxygenation-Induced PPAR*α*-mRNA Expression in Hepatocytes

**Figure 4:**
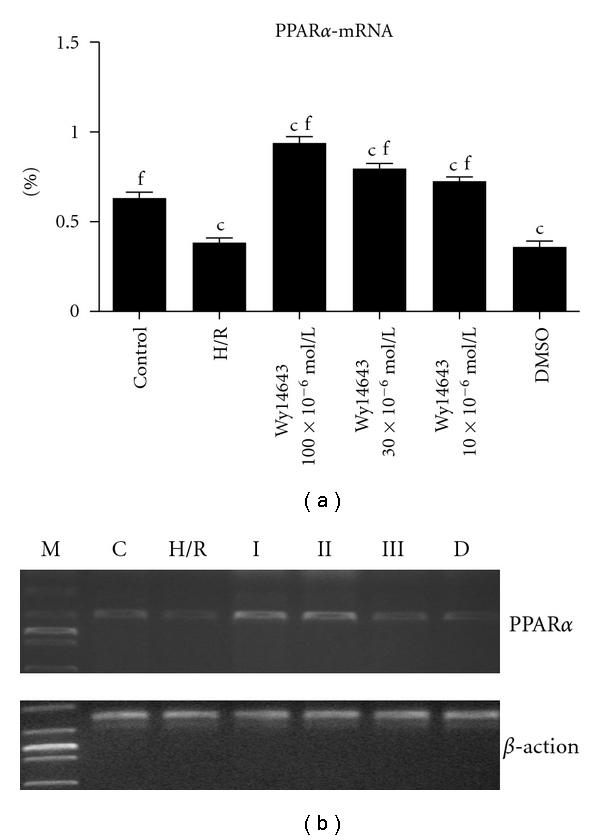
After 4 h of hepatocellular hypoxia and 10 h of reoxygenation. Effect of hypoxia and reoxygenation on PPAR*α*-mRNA expression in hepatocytes. (Mean ± SD, *n* = 6) ^c^ *P* < 0.01 versus control group; ^e^ *P* < 0.05, ^f^ *P* < 0.01 versus H/R group. (C: control group; H/R: hypoxia/reoxygenation group; I: Wy14643 group 100 × 10^−6^ mol/L; II: Wy14643 group 30 × 10^−6^ mol/L; III: Wy14643 group 10 × 10^−6^ mol/L; D: DMSO group).

PPAR*α*-mRNA expression was assessed in the absence and presence of Wy14643 during inducing hepatocytes by H/R. Quantitative analysis showed that the mRNA expressions of PPAR*α* in the H/R group was significantly decreased when compared with the control group (*P* = 0.005). However, when comparing the Wy14643 group (100 × 10^−6^, 30 × 10^−6^, 10 × 10^−6^ mol/L) with the H/R group, mRNA expressions of PPAR*α* were increased after the addition of Wy14643 (*P* = 0.0018; *P* = 0.0073; *P* = 0.0098) (Figure 4). DMSO group was not considered statistically when compared with H/R group (*P* = 0.065).

### 3.5. Ultrastructure Alterations of Hepatocytes

**Figure 5:**
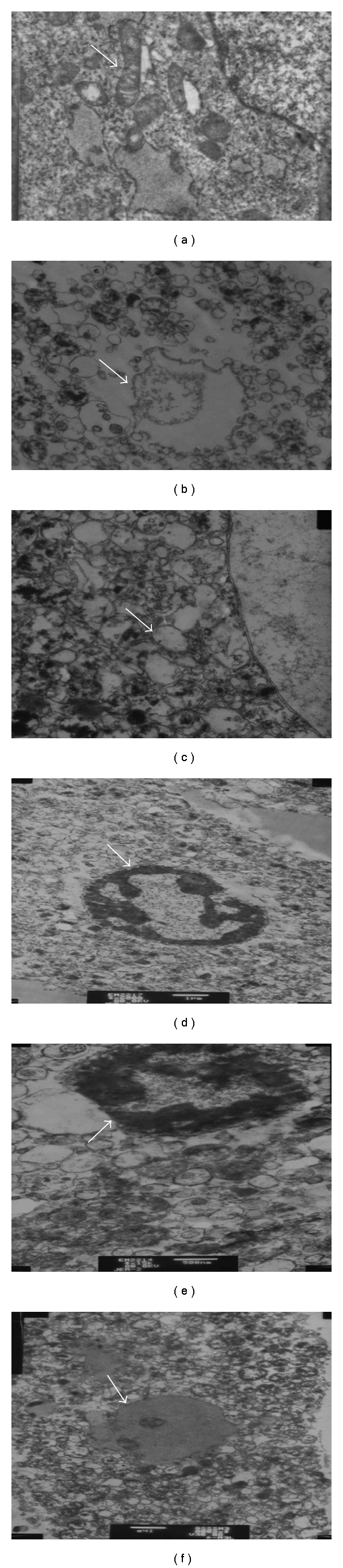
Ultrastructure alterations of hepatocytes (a) Control group, Nomal appearance of mitochondrion, nucleus struct. (X = 12 000) (b) H/R group, Mitochondrial crista destruction and nucleus structure destruction. (X = 12 000) (c) Wy14643 100 × 10^−6^ mol/L group, Nomal appearance of mitochondrion and nucleus structure almost. (X = 12 000) (d) Wy14643 30 × 10^−6^ mol/L group, Mitochondrion swellen mildly and nucleus structure are nomal nearly. (X = 12 000) (e) Wy14643 10 × 10^−6^ mol/L group, Mitochondrion swellen significantly, vacuolar degeneration and mitochondrial crista destruction, nucleus structure destruction. (X = 12 000) (f) DMSO group, Mitochondrion swellen significantly, vacuolar degeneration and mitochondrial crista destruction, nucleus structure destruction. (X = 12 000).

The ultrastructure of hepatocytes was normal in the control group (Figure 5(a)). Compared with the control group, H/R group was markedly damaged characterized by mitochondrial crista destruction markedly decreased and nucleus structure destruction (Figure 5(b)). In Figure 5(c), Wy14643 group (100 × 10^−6^ mol/L), almost normal appearance of mitochondrion and nucleus structure. In Figure 5(d), Wy14643 group (30 × 10^−6^ mol/L), Mitochondrion swelled mildly and normal nucleus structure. In Figure 5(e), Wy14643 group (10 × 10^−6^ mol/L), Mitochondrion swelled moderatedly, with mitochondrial crista interruption and nucleus structure destruction. In Figure 5(f) (DMSO group), mitochondrion swellen significantly and nucleus structure destruction.

## 4. Discussion

This study provides compelling evidence that the selective PPAR*α* agonist Wy14643 protects the hepatocytes from transient H/R injury. An increase in the cell medium's ALT and AST levels has been usually used as an effective indicator of impaired hepatocytes with H/R. The protective effect of Wy14643 demonstrated herein by reducing ALT and AST levels is associated with an inhibition of oxidative stress and upregulation of hepatocytes PPAR*α*-mRNA expression. Previous studies provided evidence that Wy14643 protected the rat liver from hepatic I/R injury [10]. In our study, we found that pretreatment of Wy14643 resulted in a marked reduction of ALT and AST levels with dose-dependent manner in the cell medium compared with the control group, as well as we demonstrated a significant down regulation in PPAR*α*-mRNA after H/R, and this downregulation was significantly attenuated by Wy14643.

The precise sequence of events leading to hepatic I/R is still a matter of debate. However, two major culprits have been identified including uncontrolled oxidative stress and unfettered inflammation, whose effects culminate in the necrotic cell death of hepatocytes, the hallmark of severe hepatic I/R [22]. Therapies aimed at curbing inflammation and blocking oxidative necrosis of hepatocytes in the setting of hepatic I/R have been heavily explored. The precise sequence of events leading to hepatic I/R has not been completely elucidated. As is well known H/R has been shown to stimulate a burst of ROS of mitochondrial and extra mitochondrial origin; as reported, ROS was generated by cytosol and mitochondria, in different cell types cultured under 1.5–5% O_2_ [23, 24]. Although hypoxia stimulates ROS generation and the contribution of mitochondria to this process may vary depending on whether reoxygenation follows the hypoxic phase, it has been reported that Wy14643 uncouples mitochondrial oxidative phosphorylation in isolated hepatic mitochondria [25]. MDA is the end product of lipid peroxidation considered as a sensitive index to assess lipid peroxidation [26]. SOD, an oxygen radical scavenger which converts superoxide anion radicals present in the upper stream of reactive oxygen metabolism cascade and protects cells against damage. The hepatic MDA levels significantly increased, indicating the presence of enhanced lipid peroxidation due to H/R injury [27], whereas the level of SOD declined, demonstrating the depletion of antioxidant pool in hepatocytes H/R. In this study we showed that Wy14643 significantly decreased MDA levels in the hepatocytes when compared with the H/R group. In addition, high levels of SOD were observed in the Wy14643-pretreated groups. According to these findings, Wy14643 has protective effects against the oxidative stress induced hepatocytes injury. This finding is in agreement with other reports showing that Wy14643 enhances expression of antioxidant enzymes such as SOD and catalase in the rat liver [28]. Our data suggests that the mechanism underlying the protective action of Wy14643 against hepatocellular H/R is the direct ROS scavenging effect.

GSH is a major cellular antioxidant which is found mainly in cytosol where it is synthesized from its constituent amino acids and in mitochondria where it plays a key protective role against oxidant-induced cell death [29]. Because of its antioxidant function, hypoxia would be expected to reduce intracellular GSH stores. The present study examined for the first time the impact of mitochondria GSH depletion on the survival of hepatocytes which were pretreated by Wy14643 during exposure to hypoxia. Previous studies reported the decrease of hepatocellular GSH stores by hypoxia. This study addressed the role of GSH, particularly in mitochondria, on the susceptibility of hepatocytes to hypoxia-induced oxidative stress. Consistent with the burst of ROS generation, it has been shown that hypoxia depletes GSH stores [30, 31]. In agreement with these findings we showed GSH depletion in both cytosol and mitochondrial compartments compared with H/R. Here, we found that Wy14643 increased the content of GSH in the hepatocytes which were induced by H/R. Our result showed that Wy14643 significantly increased the production of GSH compared with the H/R group.

In the present study, we found that H/R stress lead to decreases of PPAR*α*-mRNA in hepatocytes. A variety of stimuli, including hypobaric hypoxia, are also capable of inducing a switch in substrate use associated with downregulation of PPAR*α*-regulated genes [32, 33]. Wy14643 attenuates the increase in PARP (poly-ADP-ribose polymerase) activity caused by splanchnic artery occlusion (SAO) shock, ROS produce strand breaks in DNA, which trigger energy-consuming DNA repair mechanism and activate the nuclear enzyme PARP. PARP plays an important role in ischemia/ reperfusion injury. PARP activation results in the depletion of its substrate NAD^+^ and also in a reduction in the rate of glycolysis. As NAD^+^ functions as a cofactor in glycolysis and the tricarboxylic acid cycle, NAD^+^ depletion leads to a rapid fall in intracellular ATP rapidly followed by cellular dysfunction and death—the PARP Suicide Hypothesis. Huss et al. [33] found that hypoxia deactivates PPAR*α* by reducing the availability of its obligate partner RXR (retinoid X receptor). Mitochondrial oxidative phosphorylation is inhibited, and ATP generation is reduced, which aggravates hepatocellular anoxic injury. Hence, PPAR*α* may improve the energy supplement of hepatocytes meanwhile enhancing the anti-ischemia ability of hepatocytes. Previous data showed a rat cardiac model of hypoxia and have found a downregulation in its expression of PPAR*α* [34]. These results show, similar to our study, that the expression of PPAR*α*-mRNA in hepatocytes which were exposed to H/R was reduction. After pretreatment of Wy14643, the expression of PPAR*α*-mRNA increased; meanwhile, the damage of hepatocytes was relieving. Thus, it is conceivable; the expression of PPAR*α* during inflammation and hypoxia may serve as a counter regulator of ROS production. 

In summary, PPAR*α* agonist Wy14643 decreased the injury degree with the hepatocytes H/R injury model, which is associated with modulating the expression of PPAR*α*. Moreover, Wy14643 also protected the hepatocytes against oxidative stress. These findings are particularly interesting because they demonstrate that a regulatory factor PPAR*α* expressed in liver parenchymal cells, but not in Kupffer cells, may have significant impact on the hepatic inflammatory response. From a clinical standpoint, most of the new knowledge that we have gathered on the multiple “hepatoprotective” functions of PPAR*α* including protection from oxidative necrosis is both conceptually important and directly relevant to clinical problems associated with liver transplantation and liver disease. However, before the clinical therapeutic application of this agent, further investigations should be performed. The effects of Wy14643 on prolonged H/R injury or the effects on the chronic phase should be studied.
